# Audit of laparoscopic surgery for colon cancer in Morocco: A report of the results of a prospective multicentre cohort study

**DOI:** 10.1016/j.amsu.2022.104290

**Published:** 2022-08-03

**Authors:** Aya El Yaakoubi, Salma Lahmadi, Amine Benkabbou, Raouf Mohsine, Abdelkader Belkouchi, Tijani El Harroudi, Hadj Omar El Malki, Abdelmalek Hrora, Amine Souadka, Mohammed Anass Majbar

**Affiliations:** a National Institute of Oncology, Ibn Sina University Hospital, Rabat, Morocco; b Faculty of Medicine and Pharmacy, Mohammed V University in Rabat, Morocco; c Surgical Department A, Ibn Sina University Hospital, Rabat, Morocco; d Mohammed 6 University Hospital, Oujda, Morocco; e Surgical Department C, Ibn Sina University Hospital, Rabat, Morocco

**Keywords:** Colon neoplasms, Laparoscopy, Audit, Outcomes

## Abstract

**Background:**

Little data is available about colon laparoscopic surgery in low mid-income countries. The aim of this study was to audit the status and results of laparoscopic colon cancer surgery in Morocco.

**Patients and methods:**

This was a prospective study performed at 4 academic departments in Morocco between January 1, 2018, and March 31, 2020. All adult patients who underwent elective right or left colonic resection for colon adenocarcinoma were included. The main outcomes were the rate of laparoscopic surgery (LS) and the comparison of its short-term outcomes with open surgery (OS).

**Results:**

Among 121 patients included, 52 (43%) underwent laparoscopic resection (0–49.3%). Five surgeons (29%) performed at least one laparoscopic resection. There were more left colectomies in the laparoscopic group (71.2% vs. 39.1%. p = 0.0004), and more extended resections (23.1% vs. 40.6%. p = 0.043) and T4 stage (19% vs. 37.5%. p = 0.037) in the open group. There were no differences in 90-days overall and serious complications. OS patients had significantly more harvested lymph nodes (14 vs. 18. P = 0.007) and higher median surgical margins (6 cm vs. 9 cm. P = 0.003) than LS patients.

**Conclusions:**

LS for colon cancer in Morocco is performed by few surgeons, who apply strict patient selection for laparoscopic cases. It was associated with lower quality resections compared to open surgery. There are still many challenges requiring more focus on training, certification, centralization and standardisation of care across the nation.

## Highlights

• Laparoscopy is the standard technique for colon cancer in rich countries. Little data exists about lower income countries.
• We evaluated laparoscopy for colon cancer by comparing it to open colon resections in a low-mid income country.
• We found that Laparoscopy is performed by few surgeons, who apply strict patient selection for laparoscopic cases.
• Patients in the laparoscopy group also had lower quality resections compared to open surgery.
• The challenges identified will require more focus on training, certification, centralization, and standardisation of care.

## 1 Introduction

Several randomised control trials, systematic reviews and meta-analysis have been conducted regarding the benefits of laparoscopic surgery for colon adenocarcinoma resection. Initially, the first studies showed comparable outcomes in terms of resection and lymph node harvest suggesting equal short-term oncological outcomes. However, surgeons were first cautious in accepting laparoscopically assisted colectomies due to port site metastasis [1] and the long learning curve [2]. From 2005, randomised controlled trials (RCT) showed better short term outcomes for laparoscopic surgery, with similar oncological outcomes [3,4]. Veldkamp et al. showed that laparoscopic surgery was associated with less blood loss, earlier recovery of bowel function, less need for analgesics, and with shorter hospital stay [5]. More recently, in a Japanese RCT, Toritani at al. showed that laparoscopic surgery was associated with even better short-term health related quality of life when compared to open surgery for transverse and descending colon cancer [6]. Therefore, in developed countries such as the USA, Japan and France, laparoscopic surgery for colon cancer resection became the gold standard procedure for colon cancer [[7], [8], [9]].

In Morocco, colon cancer is the fifth most common cancer in both sexes and the first digestive cancer [10], although the incidence seems to be lower than that of western countries. According to the WHO GLOBOCAN 2020, the incidence of colon cancer in Morocco is 11.3, compared to 28.8 and 30.1 per 100 000 in Europe and the USA respectively. Moreover, the mortality rates remain high, with 6.2, 12.0 and 8.0 per 100 000 in Morocco, Europe, and USA, respectively [11].

In Morocco, as for other low- and mid-income countries (LMIC), patients are challenged by the difficult access to health care, and consequently, are diagnosed in advanced cancer stages [12,13]. Although access to basic laparoscopic procedures such as cholecystectomy remains low in most cities in the country [14], some surgeons in academic centres have shifted to laparoscopic approaches for advanced diseases such as rectal and liver cancers [15,16]. However, there is, to our knowledge, no available published studies assessing the use of laparoscopic surgery in colon cancer in LMIC. Therefore, we are reporting the first LMIC national multicentric study that aims to audit the status and results of laparoscopic colon cancer surgery in Morocco.

## 2 Material and methods

This study analysed data from the “Observatory of the Quality of Surgical Procedures for Digestive Cancers”, a multicentre observational prospective cohort study aiming to evaluate the quality of surgical care for digestive cancer patients in Morocco [17]. This study is registered at Clinicaltrials.gov under the number NCT03681600 and was approved by the Mohammed Vth University ethical committee for biomedical research in Rabat (Morocco) under the number 57/17. The study is reported according to the Recommendations for Strengthening the Reporting of cohort, cross-sectional and case-control studies in Surgery (STROCSS) statement [18]. Informed written consent was obtained from all individuals included in the study.

### 2.1 Study design

This prospective study is a subgroup analysis of patients treated for colonic cancer in 4 participating academic departments. Patients were included between January 1, 2018, and March 31, 2020. Initially, the study was scheduled to finish in December 2020, but was interrupted in March 2020 because of COVID19 pandemic [19].

### 2.2 Participants

All adult patients (16 years and above), with a histologically proven primary adenocarcinoma of the colon, and who had elective right or left colonic resection for either palliative or curative intent, were included. We excluded patients with other histological types, a transverse or total colectomy or who refused to give written consent to participate in the study. The collected data included patient's (age, sex, body mass index, major co-morbidities, American score of Anaesthesiologist and performance Status scores) and diseases characteristics (histology, TNM staging, location of the tumour), operative details (surgical procedure, surgery duration, blood loss) and postoperative outcomes.

### 2.3 Outcomes

The aim of this study was to assess the outcomes of laparoscopic surgery for colon cancer, by comparing the short-term outcomes with open colonic resection. The choice between laparoscopic and open approaches was decided by the local surgical teams. Data of patients with conversion to open surgery was analysed with the laparoscopic group (intention to treat).

Patients and disease characteristics were compared between the two groups to identify differences in patients’ selection and help to better interpret the outcomes. Primary outcomes were 90-days postoperative overall morbidity, major morbidity and the pathological analysis of the surgical specimen. Morbidity was evaluated according to the Clavien-Dindo grading system. Serious morbidity was defined as complications graded higher than grade III [20]. For the surgical specimen, the number harvested lymph nodes, surgical margins and the rate of R1 resections were analysed.

Secondary outcomes were to compare the rates of anastomotic leaks, wound infections, surgical revision and the 90-days readmission rates between the two approaches.

### 2.4 Data collection and quality control

Patients’ data was collected prospectively in paper standardised forms by the local medical teams in each department. A final independent anonymous database with electronic case report forms was filled by qualified and trained local data managers of an independent contract research organisation (CRO) [17].

Each surgical department was audited monthly by the independent CRO, with a focus on essential study documents, informed consent procedures, eligibility criteria and source data verification.

### 2.5 Statistical methods

All categorical variables presented as numbers and percentages. Quantitative variables are presented as mean (with standard deviation) or median (and quartiles) as appropriate. Comparison of quantitative variables was done using the “t” Student or the Mann-Whitney-U tests, as appropriate. Statistical significance was defined as p < 0.05.

## 3 Results

### 3.1 Description of the population

**Fig. 1:**
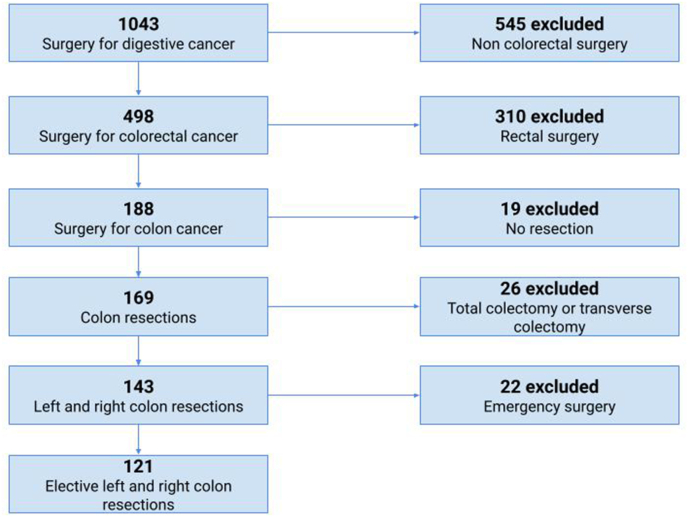
Flowchart of patients' selection.

Overall, the database included 1040 patients operated for digestive cancer. Among them, 188 underwent surgery for colon cancer. After excluding patients who underwent emergency surgery, non-resection surgery, total and transverse colectomy, the remaining 121 patients were included in the final analysis (Fig. 1). Of these, 52 patients (43%) underwent laparoscopic resection. The rate of laparoscopic surgeries performed per centre ranged from 0 to 49.3%. Among a total of 17 attending surgeons, 5 performed at least one laparoscopic colon resection (29%).

The median age was 58 years (49, 69), and male patients accounted for 66 (54.5%). Performance status score was 0–1 for 117 patients (96.7%) and the American Score for Anaesthesiologists score was 1–2 for 84 patients (69.4%). The median BMI was 23.92 (19.84, 26.73). The resection intent was curative for 117 patients (96.7%). Fifty-seven patients had a right colectomy (47.1%), and 64 patients had a left colectomy (52.9%).

### 3.2 Comparison between laparoscopic and open surgery

#### 3.2.1 Patients and disease characteristics

Overall, there were no statistically significant differences in patient's characteristics and comorbidities (Table 1). There were more left colectomies in the laparoscopic group (71.2% vs. 39.1%. p = 0.0004) group. There were more extended resections (23.1% vs. 40.6%. p = 0.043), T4 stage (19% vs. 37.5%. p = 0.037) higher rate of positive lymph nodes on surgical specimens (49.3% vs. 74.5%. P = 0.005) in the open surgery group.

**Table 1:** Comparison of patients and disease characteristics between laparoscopy and open surgery.

	Open Surgery	Laparoscopy	P value
**No. of patients (121)**	69(57.02%)	52(42.97%)	
**Age**			**0.838**
Median(quartiles)	58(48.5–70)	58(49–69)	
**Sex**			**0.814**
Male	37(53.6%)	29(55.8%)	
Female	32(46.4%)	23(44.2%)	
**Smoking**			**0.428**
Yes	5(7.2%)	2(3.8%)	
No	64(92.8%)	50(96.2%)	
**History of stroke**			**0.247**
Yes	0%	1(1.9%)	
No	69(100%)	51(98.1%)	
**Ischemic heart disease**			**0.383**
Yes	1(1.4%)	0%	
No	68(98.6%)	52(100%)	
**Diabetes**			**0.229**
Yes	2(2.9%)	4(7.7%)	
No	67(97.1%)	48(92.3%)	
**Anticoagulant treatment**			**0.848**
Yes	1(1.5%)	1(1.9%)	
No	67(98.5%)	51(98.1%)	
**PS score**			**0.21**
OMS 0-1	65(94.2%)	52(100%)	
OMS 2-4	2(2.9%)	0%	
**ASA score**			**0.664**
ASA 1-2	47(68.1%)	37(71.2%)	
ASA 3-4	21(30.4%)	15(28.8%)	
**Hb**			**0.969**
<12	47 (68.1%)	35 (67.3%)	
≥12	21 (30.4%)	17 (32.7%)	
Missing	1 (1.4%)	0%	
**Albumin**			**0.157**
<30	10(14.5%)	0%	
>30	31(44.9%)	44 (84.6%)	
**BMI**			**0.836**
<18	10 (14.7%)	4 (7.8%)	
≥18	48 (70.6%)	42 (82.4%)	
**Differentiation**			**0.786**
Well differentiated	54(78.3%)	43(82.7%)	
Other	11(15.9%)	6(11.5%)	
**Resection**			**0.773**
Curative	67(97.1%)	50(96.2%)	
Palliative	2(2.9%)	2(3.8%)	
**Surgical procedure**			**0.0004**
Right colectomy	42(60.9%)	15(28.8%)	
Left colectomy	27(39.1%)	37(71.2%)	
**Anastomosis creation**			**0.733**
Yes	67(97.1%)	51(98.1%)	
No	2(2.9%)	1(1.9%)	
**Protective stoma**			**0.253**
Yes	0%	2(3.8%)	
No	68(98.6%)	49(94.2%)	
**Associated resection**			**0.043**
No	41(59.4%)	40(76.9%)	
Yes	28(40.6%)	12(23.1%)	
**T stage**			**0.037**
pT1-T3	40 (62.5%)	32(76.2%)	
T4	24(37.5%)	8(19%)	
**N stage**			**0.005**
pN0	35(50.7%)	13(25.5%)	
pN1 or more	34(49.3%)	38(74.5%)	

PS: Performance status/ASA: American Score of anaesthesiologists/BMI: Body Mass Index/

#### 3.2.2 Comparison of outcomes

Comparison of outcomes between laparoscopy and open surgery are shown in Table 2.

**Table 2:** Comparison of outcomes between laparoscopy and open surgery.

	Open Surgery	Laparoscopy	P value
**No. of patients (121)**	69(57.02%)	52(42.97%)	
**Perioperative tumoral perforation**			**0.078**
No	64(94.1%)	51(100%)	
Yes	4(5.9%)	0%	
Missing	1	1	
**Perioperative contamination**			**0.111**
No	61(91%)	50(98%)	
Yes	6(9%)	1(2%)	
Missing	2	1	
**Bleeding**			**0.84**
No	68(98.6%)	51(98.1%)	
Yes	1(1.4%)	1(1.9%)	
**Postoperative blood transfusion**			**0.009**
No	53(76.8%)	49(94.2%)	
Yes	16(23.2%)	3(5.8%)	
**Surgical revision**			**0.383**
No	65(97%)	44(93.6%)	
Yes	2(3%)	3(6.4%)	
Missing	2		
**Wound infection**			**0.862**
No	63(91.3%)	47(90.4%)	
Yes	6(8.7%)	5(9.6%)	
**Deep fluid collection**			**0.891**
No	66(95.7%)	50(96.2%)	
Yes	3(4.3%)	2(3.8%)	
**Anastomosis fistula**			**0.402**
No	66(95.7%)	48(92.3%)	
Yes	1(1.4%)	3(5.8%)	
**Time spent in the ICU**			**0.562**
Median (min) (quartiles)	1 (0,1)	1 (0,1)	
**Higher Clavien Score**			**0.41**
< Grade II	15(21.7%)	11(21.2%)	
> Grade II	7(10.1%)	2 (3.8%)	
**Duration of stay**			**0.634**
Median (quartiles)	7 (6,10)	7 (6,8)	
**Unprogrammed hospital readmission within 90 days**			**0.706**
No	63(94%)	44(95.7%)	
Yes	3(4.5%)	2(4.3%)	
**Clavien Score within 90 days**			**0.915**
< Grade II	59 (85.5%)	43 (82.7%)	
> Grade II	10(14.5%)	9 (17.3%)	
**Margins**			**0.003**
Median (quartiles)	9 (6.63, 11.38)	6 (4.4, 9.75)	
**No. of ganglions harvested**			**0.007**
Median (quartiles)	18 (12, 27)	14 (11, 18)	
**Radicality**			**0.151**
R1	3 (4.8%)	0%	

##### 3.2.2.1 Intraoperative outcomes

The median duration of surgery was higher in the LAP group (200 min vs. 180 min. P = 0.052) and there was more blood loss in the OC group (50 ml vs. 100 ml 0.08). LAP was associated with no tumour perforation (0% vs. 5.9%. p = 0.078), and less perioperative contamination (2% vs. 9%. p = 0.111).

##### 3.2.2.2 Postoperative outcomes

There were no differences in 90-days overall and serious postoperative complications (14.5% vs. 17.3%. p = 0.91) between the two groups. Wound infection (8.7% vs. 9.6%. P = 0.86), anastomotic leak (1.4% vs. 5.8%. p = 0.402) and surgical revision rates (3% vs. 6.4%. p = 0.38) were not different between the two groups. However, LAP group patients had less postoperative blood transfusion (5.8% vs. 23.2% p = 0.009). There wasn't a difference in terms of mortality between the two groups (0% vs. 1.4%). Finally, the duration of stay (7 vs. 7 days. p = 0.63), unplanned hospital readmission within 90 days (4.3% vs. 4.5%. P = 0.706) were not different between the two groups.

#### 3.2.3 Pathology outcomes

OC patients had significantly more harvested lymph nodes than LAP patients (14 vs. 18. P = 0.007) and higher median surgical margins (6 cm vs. 9 cm. P = 0.003). No patients were recorded to have R1 resection in the LAP group, compared to 3 patients in the OC group (0% vs. 4.8%. P = 0.151).

## 4 Discussion

This National prospective multicentric study aimed to assess the use and the short-term outcomes of laparoscopic colonic resection for cancer in Morocco. The rate of laparoscopic colectomies was 43%, performed by 5 surgeons among 17 (29%). The rate of laparoscopic resections was different between centres, ranging from 0 to 49%. The results showed no differences in the short-term postoperative complications and hospital stay. However, the quality of resection was significantly better in the open surgery group.

Laparoscopic colon surgery was associated with better patient selection in terms of patients and disease characteristics. Comparing laparoscopic and open groups, patients in the laparoscopic group had significantly lower BMI, higher albumin levels, and less T4 tumours. Also, there were more left colectomies than right laparoscopic colectomies. These results indicate that there was a strict patients’ selection by surgeons performing laparoscopic procedures.

There are many challenges in introducing mini-invasive techniques in mid-low-income countries [[21], [22], [23]]. High cost of equipment for a start-up in LMIC is one of the most significant barriers. Many LIMCs have poor funding of public hospitals. This leads to laparoscopic training being a privilege for some university hospitals and not widely available across the nation, such is the case in Morocco. Additionally, in Morocco, insurance reimbursement is the same for open and laparoscopic colectomies, which does not help to cover the high initial cost of implementing laparoscopic procedures. Another challenge is equipment maintenance, which is especially challenging due to the lack of funding and mishandling of the equipment [24]. Training is also a significant obstacle for performing advanced laparoscopic procedures. In Morocco, training in universities is focused on basic laparoscopic skills, and there is no specific training in advanced laparoscopic procedures [25]. Surgeons performing advanced mini-invasive techniques got their training in Europe, mostly at their own expenses [26]. This explains the low number of surgeons performing laparoscopic colectomies and the variability of using laparoscopy between centres.

This study highlighted unexpected results comparing laparoscopic versus open colectomy in the Moroccan setting. The main difference was in the quality of resection in favour of the open surgery group. Most of the previous comparative studies have shown similar quality resection between laparoscopic and open surgery [[27], [28], [29], [30]]. This difference could not be explained by patients or disease characteristics, since in the LS group there were significantly more favourable features compared to the OS group. One explanation may be the lack of experience of Moroccan surgeons in laparoscopic colectomies. In the literature, it is reported that between 30 and 70 are necessary to complete the learning curve for colon resections [31,32]. However, by performing only 52 laparoscopic resections during two years in the 4 participating centres, Moroccan surgeons did not achieve the sufficient workload to achieve competency, as recommended in the literature.

An additional unexpected result was the similar duration of stay in the two groups. In most reported western studies, laparoscopy is associated with shorter hospital stay. This result can only be explained by the social nature of cancer care in university hospitals in Morocco. Often, patients treated in university hospitals come from remote or rural areas, where continuity of surgical care may not be available. Therefore, patients cannot be discharged early to ensure that any postoperative complications can be dealt with immediately.

In order to improve the quality of surgical oncological results and to correct the low adoption of laparoscopic surgery, focus should be given to implementing advanced laparoscopic techniques training programs in Morocco. This may require in the beginning collaborations with high volume international centres to ensure adequate surgical training for Moroccan surgeons. Centralization may also be a suitable solution to the differences in standard of care in colon cancer management. Considering the low incidence of colorectal cancer in Morocco, centralization may ensure an adequate caseload to complete the learning curve in laparoscopic colon surgery, and to maintain a good quality of surgical care [[33], [34], [35]]. More focus should be shed on surgeon qualifications, and in forming a qualification assessment and certification for laparoscopic care in colorectal surgery. The Japanese ESSQS-QS had led to good short-term outcomes, less intraoperative complications and less rate of conversion [36]. Similar accreditation systems were implemented in Thailand [23] as well as other programs for continuing professional development in the UK, USA and across Europe [37]. Such approaches must be considered in Morocco to improve and maintain the quality of surgical care.

This study has two main strengths. First, to our knowledge, this is the first multi-centre national study auditing laparoscopic surgery for colon cancer in Africa. In addition, there are 5 university hospitals in Morocco, and the study included surgical departments from 2 university hospitals that are the most advanced in colorectal cancer surgery in the country [15,38,39] [40]. Therefore, we can reasonably consider the results of this study representative of surgical practice within public university hospitals in the country.

This study has several limitations. Despite the multicentric design of the study, the number of colonic resections was very low compared to similar studies in the literature. In fact, like other countries in Africa, Morocco has a low incidence of colonic cancer (4.3 per 100.000) compared to western and eastern countries, where the incidence is above 15 or 20 per 100.000 [41]. Furthermore, most patients are diagnosed at advanced stages, requiring an extended open surgery and then not fit for laparoscopic approach, or not amenable to surgical curative treatment [10] [26]. Second, the non-randomised aspect of this study may add a significant bias in patients’ selection. Furthermore, this study concerned only the academic public hospitals without the academic private institutes and private clinics. The private hospitals may have performed more laparoscopic surgeries and face fewer challenges compared to the public sector.

In conclusion, this national audit revealed that laparoscopic surgery for colon cancer in Morocco was performed by few surgeons, who apply strict patient selection for laparoscopic cases. It was associated with lower quality resections compared to open surgery in this multicentric real life data in Morocco. Improvement will require more focus on implementing advanced laparoscopic training programmes and highlight the importance of certification, centralization and standardisation of care across the nation.

## Ethical approval

This study was approved by the Mohammed Vth University ethical committee for biomedical research in Rabat (Morocco) under the number 57/17.

## Funding

This research project was funded by “L'institut de recherche sur le cancer”, Fes, Morocco (www.irc.ma). Project number 201951/AP2016.

## Authors contributions

El Yaakoubi Aya (Manuscript drafting, statistical analysis), Lahmadi Salma (Manuscript drafting, statistical analysis), Benkabbou Amine (Study design, manuscript review)), Mohsine Raouf (Data collection), Belkouchi Abdelkader (Data collection), El Harroudi Tijani (Data collection), El Malki Hadj Omar (Data collection), Hrora Abdelmalek (Data collection), Souadka Amine (Data collection, statistical analysis, manuscript writing), Majbar Mohammed Anass (Study design, Data collection, statistical analysis, manuscript writing).

## Consent

Written consent was obtained from each patient before the inclusion in the study.

## Registration of research studies

1. Name of the registry: ClinicalTrial.gov
2. Unique Identifying number or registration ID: NCT03681600
3. Hyperlink to your specific registration (must be publicly accessible and will be checked): https://clinicaltrials.gov/ct2/show/NCT03681600

## Provenance and peer review

Not commissioned, externally peer-reviewed.

## Guarantor

Prof Majbar Mohammed Anas.

## Declaration of competing interest

All authors declare no conflict of interest.
